# Tobacco-alcohol optic neuropathy – clinical challenges in diagnosis

**Published:** 2014

**Authors:** SM Chiotoroiu, M Noaghi, GI Stefaniu, FA Secureanu, VL Purcarea, M Zemba

**Affiliations:** * “Nicolae Malaxa” Clinical Hospital, Bucharest; ** “Carol Davila” University of Medicine and Pharmacy, Bucharest; *** Clinical Ophthalmological Emergency Hospital

**Keywords:** optic neuropathy, tobacco, alcohol, toxins, visual field

## Abstract

Part of the large group of nutritional and toxic optic neuropathies, tobacco-alcohol optic neuropathy is a disease often underdiagnosed or detected at a stage when the full recovery of vision is not possible. This article summarizes its signs and symptoms, describes the pathophysiological processes involved and provides the necessary information for diagnosis and treatment of the entity previously known as tobacco-alcohol amblyopia, reporting in the end, a challenging case along with its findings.

## Introduction

Nutritional and toxic optic neuropathies (NTON) represent a group of medical disorders defined by visual disturbances due to optic nerve damage by a nutritional deficit or a toxin. It has previously been suggested that certain foods, toxins and neurotrophic drugs can interfere with the physiological processes in the retinal cells and synapses of the afferent visual pathway controlled by specific neurotransmitters, proteins and enzymes.

Each component of the afferent visual pathway is susceptible to the effects of drugs, toxin or nutritional factors resulting in visual impairment that can be expressed subjectively by the patient or encountered through certain visual function tests [**1**]. According to Glaser, both males and females are equally involved, all ages can be affected and there is no predilection for races [**2**].

The most common form of NTON is related to the chronic use of alcohol in heavy smokers, followed in frequency by the ones associated with the use of medication such as ethambutol, amiodarone and chloroquine [**3**].

**Semiology**

Often underdiagnosed or detected at a stage when full recovery of vision is not possible, nutritional and toxic optic neuropathies have a similar clinical presentation. Commonly, they display simultaneously and bilaterally. The visual decline is usually progressive, bilateral, and symmetrical without any pain. However, most authors consider that dyschromatopsia is frequently the first symptom with the majority of patients noticing that certain colors, mostly red, lose their brightness [**4**,**5**]. These disturbances in color vision often foresee the loss of visual acuity which starts as a gradual progressive blurriness at the point of fixation and declines in a continuous pattern to values of 20/400 (0.05) or above, except for the case of methanol intoxication when complete blindness with no perception of light may occur [**4**].

At the slit-lamp examination, the pupillary light response may be normal, bilaterally sluggish or absent for those nearly or completely blind. The optic disc is usually normal in the early stages, swollen or mildly hyperemic with splinter hemorrhages later. Disc edema and hyperemia are frequently seen in acute intoxications. With the progression of the disease, temporal disc pallor with papillomacular bundle atrophy may be observed. In the final stages, extended damage to the nerve fibers results in total optic atrophy [**6**]. However, the primary lesion is this group called optic neuropathies has not actually been localized with certainty in the optic nerve fibers and may originate in the retina, the optic chiasm or even the optic tracts [**7**].

The visual field findings are usually bilateral, symmetric, with preservation of the peripheral field and include:

1. Bilateral cecocentral scotoma. This defect extends from the physiological “blind spot” through and into the point of fixation. Although a central scotoma may also encompass the blind spot, a cecocentral scotoma is smaller and typically, dumbbell shaped.

2. Bilateral central scotoma. This involves the central 5° to 20° surrounding fixation. Central scotoma usually indicates damage to the macular retinal ganglion cells or to the papillomacular nerve fibers at or within the optic nerve. If the scotoma incorporates the blind spot, the optic nerve is certainly involved [**8**].These defects usually have soft margins. Rarely, other types of visual field injuries have been reported: paracentral scotomas - these involve defects within 20° of the fovea, diffuse depression - there is generalized decreased visual sensitivity.

**Pathophysiology**

Common etiologies include malnutrition, toxic exposure (ethanol, methanol, tobacco, chloramphenicol, ethambutol, isoniazid, streptomycin, amiodarone, digitalis, chloroquine, disulfiram, lead, and others), and vitamin deficiency (B group: thiamine, cyanocobalamin, riboflavin, pyridoxine, folic acid) usually acquired after chronic exposure to alcohol and tobacco. For the majority of these entities, the cause is represented by the impairment of the vascular supply of the optic nerve and the metabolism through the toxic substances or their metabolites. Their mechanisms of action result in a negative impact on the mitochondrial oxidative phosphorylation.

For example, for the tobacco-alcohol optic neuropathy (also known as tobacco-alcohol amblyopia) the mechanism is still unclear. However, vitamin B12 and folate deficiencies and the cyanide in tobacco may lead to the demyelination of the optic nerve [**9**]. In addition, cyanide and free radicals from tobacco are believed to impair the mitochondrial respiratory cycle [**10**], damage the DNA [**11**] and lead to pathological changes even in the mitochondrial morphology [**12**].

Through metabolic acidosis and formate, methanol blocks mitochondrial pathways in the retina and optic nerve causing focal retrolaminar optic nerve delamination [**13**]. For ethambutol, its chelating properties have been implied to play a significant role in its toxicity due to a calcium influx into the mitochondria and excitotoxicity [**5**,**14**].

**Evaluation**

A detailed medical history accompanied by a thorough eye examination provides enough information to make a presumptive diagnosis of NTON. A complete blood cell count with peripheral blood smear examination and MRI of brain and orbit should be required for differential diagnosis. Further testing in order to establish the cause is guided by present results (blood testing for methanol, serum B12, red cell folate levels, vitamin assays, urinalysis, heavy metal screening).

Visual field testing by static or kinetic perimetry is absolutely essential from the beginning while the OCT proves its efficacy only in the later stages, failing to detect changes in the early phases [**15**]. The visual evoked response, P100 wave amplitude, was found to be markedly reduced with normal to near normal latency in patients with tobacco alcohol amblyopia [**16**]. Contrast sensitivity measurement and testing hue discrimination could also detect the early damage to the nerve fibers.

**Management**

The most important measure in approaching nutritional and toxic neuropathy is represented by the removal of the causative agent. After this first step, a significant improvement in the visual function can usually be objectified.

Besides the toxins in tobacco-alcohol neuropathy, it has been proved that the patient’s nutritional state is also of considerable importance for the recovery; most of them being in a poor nutrition state at the time of diagnosis. The cessation of smoking and alcohol use should therefore be accompanied by medical therapy with vitamin supplementation. Oral maintenance replacement therapy of thiamine, folic acid, cyanocobalamin may be appropriate for those with additional deficiencies.

Hydroxocobalamin injections used for a few weeks have proved their efficiency in visual recovery for patients affected by the Cuban epidemic neuropathy, due to the conversion of free cyanide to cyanocobalamin by hydroxocobalamin [**17**].

The discontinuation of tobacco use and drinking, establishing a well-balanced diet (green vegetables, fruits) and ensuring this lifestyle is maintained in the upcoming future by taking into account the necessary prophylactic measures that prove to be critical for the visual improvement.

The ophthalmologic examinations should be initially performed at every 3 to 4 weeks and then, depending on the patient’s visual state, at every 6 to 9 months. The visual acuity, examination of the anterior pole and retina, of the color vision should be assessed at each visit. Bilateral visual field examinations should also be completed at least once a year. Recovery of visual function may take several weeks to months although residual permanent damage to the optic nerve may sometimes persist.

Further on, a case of tobacco-alcohol optic neuropathy and its findings after OCT evaluation and standard automated perimetry testing with a Humphrey field analyzer was reported.

**Case report**

A 62-year-old female patient was referred to our service for confirmation of a presumptive diagnosis of primary open-angle glaucoma with bilateral intraocular pressure within normal range. Her medical history revealed a decreased visual acuity with a gradual onset diagnosed and investigated back in 1986, and neither progress, nor improvement over the last 20 years. She smoked one pack of cigarettes per day and drank two beers daily for 30 years before cessation of these habits 10 years ago. She denied the use of any medication on a prolonged period and, besides the visual impairment, her health state had been excellent. Her family history had no significant remarks.

Visual acuity was of 20/60 fc nc (0.3) for the right eye and 20/80 fc nc (0.25) for the left one. Intraocular pressure was within normal limits: 12 mmHg for the right eye and 15 mmHg for the left one. At the examination on the anterior segment, only limited cortical lens opacities in the left eye were observed - normal aspect for a matching-age control group. The patient’s pupils had a normal response to light stimulation with no afferent defect. Ocular motility was bilaterally normal. Fundoscopic examination displayed abnormal optic nerves with bilateral pallor of the optic nerve head, with a vertical cup/disk ration of 0,3, a modified macular area with loss of normal reflex and discrete pigmentary alterations, slightly dilated retinal veins, spastic arteries with an increased parietal reflex.

**Fig. 1:**
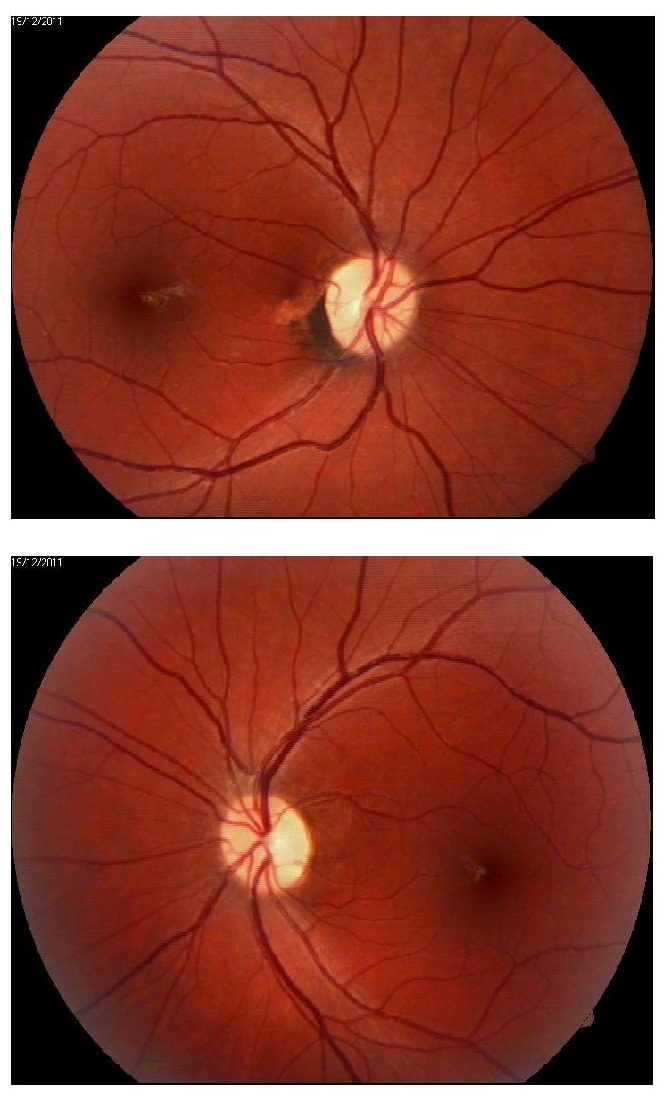
Bilateral disc pallor in a 62-year-old woman with tobacco-alcohol syndrome

Standard automated perimetry by using a Humphrey field analyzer showed a diffuse decrease in the sensitivity to light stimuli with 15.56 dB compared to the normal mean value for the age-matched controls and a concentric visual field narrowing with 15° degrees from the fixation point in the superior and inferior quadrants and up to 20° degrees in the temporal and nasal quadrants for the right eye. The examination of the visual field in the left eye also revealed a broad decrease in sensitivity to light stimuli with 17.70 dB compared to healthy age-matched subjects and a concentric narrowing up to 20° degrees nasally, 15° degrees inferiorly and 10° degrees temporally and superiorly.

There is a significant improvement in the visual fields compared to the initial situation in 1986 and this is probably due to the removal of toxins and the adoption of a healthy nutritive behavior. The examination of the visual field is of critical importance not only for the positive diagnosis of this toxic neuropathy, but also for the follow-up through consequent visits and analysis. In this manner, the efficacy of therapy and the patient’s compliance to the treatment measures can be evaluated.

**Fig. 2,3:**
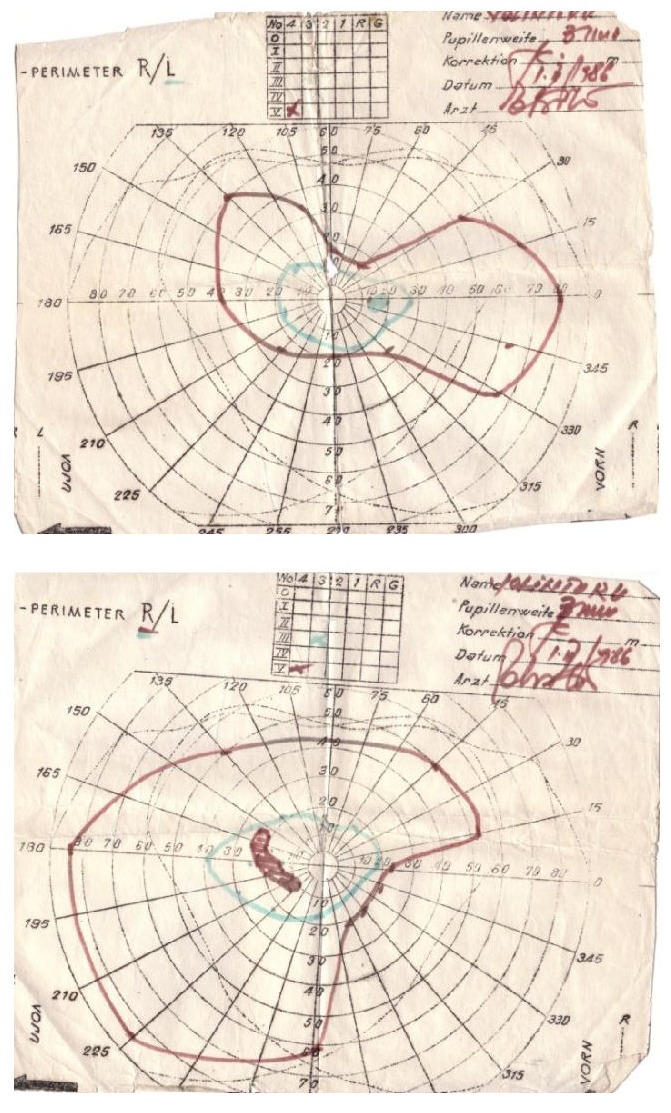
Static Goldman perimetry examinations taken at the onset of the disease, back in 1986

**Fig. 4,5:**
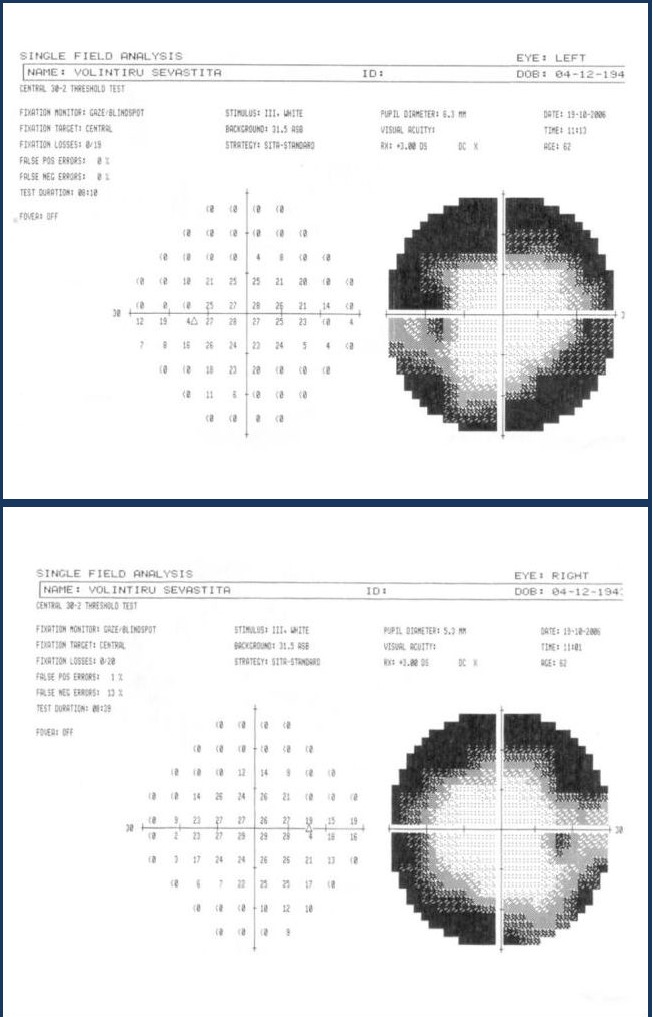
Standard automated perimetry using the central 30-2 threshold test showing concentric narrowing in both eyes

The optical coherence tomography performed with a Stratus OCT unit exposed a mild loss of the retinal nerve fiber layer; its pattern does not respect the ISNT rule in glaucoma in any way.

**Fig. 4:**
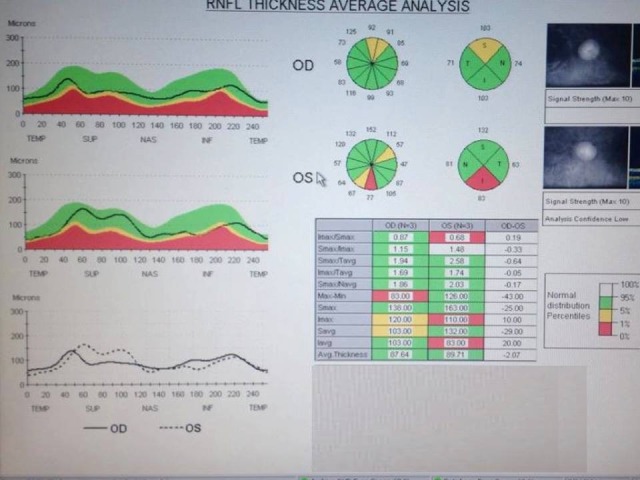
OCT image analyzing the average of RNFL thickness with a mild loss in this specific layer

The patient was also investigated through electroretinogram testing which showed a marked reduction in amplitude mostly in the peripheral regions than centrally in both eyes.

A complete blood cell count with peripheral blood smear was performed along with serum vitamin B12 and folate for differential diagnosis and they were all in normal limits. Serology for Lyme disease and antinuclear antibodies were ordered with the same purpose and they were both negative. For dismissing etiologies like ischemic or compressive syndromes, a CT scan of the brain and orbit was necessary and the results displayed no pathological changes.

## Discussions

This particular case was chosen because it stood aside from the general clinical findings and trends that were encountered in a tobacco-alcohol neuropathy and slightly resembled findings of glaucoma disease. Even though the literature described centrally impaired visual field with central or cecocentral scotomas [**4**,**18**] as classic findings for this entity, our patient had a visual field result that guided the clinician towards the diagnosis of glaucoma, but the fact that it remained in the same stage for more than a decade without treatment argued against this disease.

The discontinuation of smoking and drinking for the last 10 years most certainly played a decisive role in the current state of visual function. It may be assumed that, probably, if these measures had been taken at the first visit in 1986, the visual recovery would have been meaningful. Though, her visual problems slightly improved with time and intervention, she persisted with residual deficits.This might be due to a certain level of irreversibility of damage over time/severity of affection or a mechanism other than pure nutritional deficiency being responsibile for these dysfunctions [**19**].

All these taken into account, the diagnosis established was tobacco-alcohol toxic optic neuropathy and the treatment plan consisted of permanent cessation of vices, vitamin supplementation in the form of multivitamin tablets, dietary advices on increasing the intake of green leafy vegetables and daily fruits.

She was given adequate psychotherapy aimed at abstinence and relapse prevention and subsequently anticraving agents were instituted to reduce the possibility of relapse in future. She expressed adequate motivation for future abstinence. She has been given sheltered appointment to avoid work which required skillful visual function. But for her residual limitation in vision, she is gainfully employed.

## Conclusions

Even with low incidence and prevalence in the 21st century, the tobacco-alcohol optic neuropathy, is accompanied by a challenging clinical appearance that can create confusion among specialists. The morbidity of these disorders depend on the associated risk factors, the underlying etiology and the period of time between the first changes noticeable in the visual function and the institution of treatment. Patients with normal fundoscopy may easily recover while advanced optic atrophy is less likely.

The tobacco-alcohol neuropathy usually has a variable prognosis, essentially depending upon the nature of the agent involved, degree of exposure prior to the removal and quantity of visual acuity at the time of diagnosis.
